# Genotype, extrapyramidal features, and severity of variant ataxia‐telangiectasia

**DOI:** 10.1002/ana.25394

**Published:** 2019-01-29

**Authors:** Katherine Schon, Nienke J.H. van Os, Nicholas Oscroft, Helen Baxendale, Daniel Scoffings, Julian Ray, Mohnish Suri, William P. Whitehouse, Puja R. Mehta, Natasha Everett, Leonardo Bottolo, Bart P. van de Warrenburg, Philip J. Byrd, Corry Weemaes, Michel A. Willemsen, Marc Tischkowitz, A. Malcolm Taylor, Anke E. Hensiek

**Affiliations:** ^1^ Ataxia Telangiectasia Service, Respiratory Support and Sleep Centre Papworth Hospital Cambridge United Kingdom; ^2^ East Anglian Medical Genetics Service Cambridge University Hospitals NHS Foundation Trust Cambridge United Kingdom; ^3^ Departments of Neurology & Pediatric Neurology, Donders Institute for Brain, Cognition and Behaviour Radboud University Medical Centre Nijmegen The Netherlands; ^4^ Department of Radiology Cambridge University Hospitals NHS Foundation Trust Cambridge United Kingdom; ^5^ Department of Neurophysiology Cambridge University Hospitals NHS Foundation Trust Cambridge United Kingdom; ^6^ Department of Neurology Cambridge University Hospitals NHS Foundation Trust Cambridge United Kingdom; ^7^ Nottingham Clinical Genetics Service National Paediatric Ataxia‐Telangiectasia Clinic Nottingham United Kingdom; ^8^ School of Medicine University of Nottingham, Queen's Medical Centre Nottingham United Kingdom; ^9^ Department of Paediatric Neurology Nottingham Children's Hospital, Nottingham University Hospitals NHS Trust Nottingham United Kingdom; ^10^ Department of Medical Genetics Cambridge Biomedical Campus Cambridge United Kingdom; ^11^ The Alan Turing Institute British Library London United Kingdom; ^12^ MRC Biostatistics Unit University of Cambridge, Cambridge Biomedical Campus Cambridge United Kingdom; ^13^ Institute of Cancer and Genomic Sciences, College of Medical and Dental Sciences University of Birmingham Birmingham United Kingdom; ^14^ Department of Pediatrics Radboudumc Amalia Children's Hospital Nijmegen The Netherlands

## Abstract

**Objective:**

Variant ataxia‐telangiectasia is caused by mutations that allow some retained ataxia telangiectasia‐mutated (ATM) kinase activity. Here, we describe the clinical features of the largest established cohort of individuals with variant ataxia‐telangiectasia and explore genotype‐phenotype correlations.

**Methods:**

Cross‐sectional data were collected retrospectively. Patients were classified as variant ataxia‐telangiectasia based on retained ATM kinase activity.

**Results:**

The study includes 57 individuals. Mean age at assessment was 37.5 years. Most had their first symptoms by age 10 (81%). There was a diagnostic delay of more than 10 years in 68% and more than 20 years in one third of probands. Disease severity was mild in one third of patients, and 43% were still ambulant 20 years after disease onset. Only one third had predominant ataxia, and 18% had a pure extrapyramidal presentation. Individuals with extrapyramidal presentations had milder neurological disease severity. There were no significant respiratory or immunological complications, but 25% of individuals had a history of malignancy. Missense mutations were associated with milder neurological disease severity, but with a higher risk of malignancy, compared to leaky splice site mutations.

**Interpretation:**

Individuals with variant ataxia‐telangiectasia require malignancy surveillance and tailored management. However, our data suggest the condition may sometimes be mis‐ or underdiagnosed because of atypical features, including exclusive extrapyramidal symptoms, normal eye movements, and normal alpha‐fetoprotein levels in some individuals. Missense mutations are associated with milder neurological presentations, but a particularly high malignancy risk, and it is important for clinicians to be aware of these phenotypes. **ANN NEUROL 2019;85:170–180.**

Ataxia‐telangiectasia is a rare autosomal‐recessive disorder caused by mutations in the *ATM* gene on chromosome 11q22.3 (MIM 208900).^1^ The ataxia telangiectasia‐mutated (ATM) protein is a serine‐threonine protein kinase, which phosphorylates more than 700 substrates and is a key player in the cellular response to double‐stranded DNA damage.^2^ The clinical and genetic features of ataxia‐telangiectasia vary, and two forms of the disease have been described.

Classic (or typical) ataxia‐telangiectasia presents with a severe phenotype and has an estimated incidence of 1 in 300,000.^3^ Individuals with classic ataxia‐telangiectasia have absent ATM kinase activity,^4^ either attributed to two null mutations or mutations which result in protein without ATM kinase activity. It is a multisystem neurodegenerative disease which also causes immunological defects, respiratory problems, oculocutaneous telangiectasia, and an increased risk of malignancy.^5^, ^6^ Affected children are usually wheelchair bound before teenage years and have severe neurological disability, including cerebellar ataxia, extrapyramidal features, oculomotor dyspraxia, and polyneuropathy.^7^ Most individuals with classic ataxia‐telangiectasia die before the age of 30; malignancy or respiratory failure are the main causes of death.^8^, ^9^

In addition to classic ataxia‐telangiectasia a second form, variant ataxia‐telangiectasia has been described as a cause of neurological dysfunction.^10^, ^11^ A study of 51 patients with ataxia‐telangiectasia (including 9 with retained ATM kinase activity) showed that individuals with retained ATM kinase activity have a milder neurological phenotype.^4^ Variant ataxia‐telangiectasia results either from leaky splice site mutations which allow expression of some normal ATM protein or missense mutations which produce a mutant ATM protein with activity.^12^ It has been speculated that the presence of some ATM kinase activity relates to a milder neurological phenotype and a lower risk of systemic complications. Furthermore, a range of atypical neurological presentations have been reported to occur in some individuals with variant ataxia‐telangiectasia.

Previously published reports of variant ataxia‐telangiectasia are limited to small cohorts of less than 15 individuals^7^, ^13^, ^14^, ^15^, ^16^ or case studies of unusual presentations.^17^, ^18^, ^19^, ^20^, ^21^

The true clinical spectrum of variant ataxia‐telangiectasia is unknown, and it is not clear which factors determine the extreme clinical variability that has been reported. It is unclear whether affected individuals require specific surveillance for systemic complications and malignancy, similar to existing management recommendations of classic ataxia‐telangiectasia.^22^, ^23^

Here, we report the clinical features of the largest established cohort of patients with variant ataxia‐telangiectasia. We provide prognostic information and explore genotype‐phenotype correlations to inform management guidelines.

## Patients and Methods

### *Probands*

Clinical data were retrospectively collected from case notes for all individuals with variant ataxia‐telangiectasia who have attended the National Adult Ataxia‐Telangiectasia service (Papworth Hospital, UK), the Ataxia‐Telangiectasia Specialist Centre (Nottingham City Hospital, UK), and the Excellence Centre of Movement Disorders (Radboud UMC, Nijmegen, Netherlands). Analysis includes single cross‐sectional data using the most recent recorded clinical assessment.

### *Molecular Genetics Studies*

Patients were classified as having variant ataxia‐telangiectasia based on retained ATM kinase activity. Patients with a mutation in the initiator methionine codon were included because of some uncertainty of ATM activity associated with these mutations. *ATM* mutations were identified by Sanger sequencing of PCR‐amplified ATM exon sequences. A lymphoblastoid cell line was derived from each patient's blood, and immunoblotting for ATM expression and ATM activity assays were performed using methods as previously described.^24^ Chromosomal radiosensitivity was measured following exposure to 1Gy of gamma rays at cell cycle phase G2.

### *Neurological Assessment*

Clinical neurological assessment was performed by a neurologist with a special interest in ataxia‐telangiectasia. The Scale for Assessment and Rating of Ataxia (SARA)^25^ and Ataxia‐Telangiectasia Neurological Examination Scale Toolkit (A‐T NEST) scores^11^ were recorded. As part of the neurological examination, a subjective assessment of eye movement abnormalities was recorded by the examining neurologist as normal eye movements, mildly abnormal eye movements (including nystagmus, slowing of saccades, and mild oculomotor dyspraxia), or severely abnormal eye movements (including marked oculomotor apraxia).

### *Neurological Phenotype*

Individuals were categorized into three neurological phenotypic groups to reflect those neurological symptoms, which have been reported to most commonly occur in variant ataxia‐telangiectasia:

Group A: Cerebellar ataxia and/or peripheral neuropathy with minimal or no extrapyramidal involvement

Group B: Cerebellar ataxia and/or neuropathy plus additional extrapyramidal features

Group C: Extrapyramidal signs without significant ataxia and/or peripheral neuropathy

### *Cross‐sectional Clinical Neurological Disease Severity*

An overall assessment of current severity was made using level of mobility and self‐care.

Mild: Individuals still ambulant with/without a walking aid and could use the upper limbs for most activities without help. A‐T NEST scores within this group were > 55, SARA scores <22.

Moderate: Individuals used a wheelchair frequently, but were able to transfer, walk a few steps with help, and use the upper limbs for most activities, such as feeding themselves. A‐T NEST scores within the moderate group were 42 to 75, SARA scores 15 to 33.

Severe: Individuals were permanently wheelchair bound, were unable to transfer unaided, and had significant limitation of upper limb function, requiring assistance with most activities of daily living. A‐T NEST scores within this group were < 46, SARA scores >30.

### *Neurological Investigations*

Results of nerve conduction studies and electromyogram (EMG) were reviewed. Neuropathy was graded depending on the severity of the nerve conduction abnormality and of any accompanying EMG impairment. The neuropathy grade was determined by amplitude measures and how widespread the abnormalities were. For example, in the case of the sensory changes:

- impairment of sural amplitude only: mild
- absent sural responses: mild‐moderate
- impaired upper limb digital potential and absent sural responses: moderate
- impaired/absent upper limb potential and impaired radial amplitude: moderate‐severe
- absent sensory responses in upper limb and lower limb: severe.

A similar approach was adopted for motor potentials. If nerve conduction studies were relatively preserved, while EMG abnormality was detected, then the subcategorization of a suspected motor neuronopathy was documented.

MRI brain scans were conducted to rate atrophy of the cerebellar vermis and hemispheres on sagittal three‐dimensional T1‐weighted sequence. Cerebellar atrophy was assessed separately for the vermis and jointly for both hemispheres and rated as absent, mild (mild widening of sulci and fissures, normal size of folia), moderate (moderate widening of sulci and fissures associated with volume loss of folia), or severe (marked widening of sulci and fissures with marked volume loss of folia). White matter hyperintensities on fluid‐attenuated inversion recovery sequence were classified as punctate, beginning confluent or confluent.^26^ Cerebral microbleeds were assessed on susceptibility‐weighted imaging (SWI) or T2*W‐gradient echo sequence.^27^

#### *Respiratory Assessment*

Patients attending Papworth were assessed by a consultant respiratory physician. They underwent arterial blood gas measurement, pulmonary function testing, overnight oximetry and low dose computed tomography (CT) thorax. Five of the Dutch patients had spirometry.

#### *Immunological Assessment*

Immunological investigations included immunoglobulin levels (immunoglobulin IgG, IgA, IgM, IgG2, and IgE), serum electrophoresis if indicated, lymphocyte subset analysis, and assessment of serum antibody to pneumococcus, tetanus, and haemophilus vaccine. History of immunizations, infections, and treatment was recorded.

### *General Assessment*

Past medical history, including diabetes and malignancy, was documented in all patients. Level of mobility and age at first wheelchair use were recorded. Examination for conjunctival telangiectasia, measurement of weight, height, and body mass index (BMI), blood tests for alpha‐fetoprotein (AFP), and liver function tests (LFTs) and liver ultrasound scan were carried out in the majority of patients, but this was not standardized between centres.

### *Ethical Considerations*

The study was approved by the Health Research Authority.

### *Statistical Analysis*

In order to test the effect of the genetic covariates (presence of a missense mutation with retained ATM kinase activity, number of mild mutations, ATM protein levels, and chromosomal radiosensitivity) on neurological and other clinical features, we corrected the regression models for fixed (age, sex, and age of onset) and random effects (hospitals and families). We tested the effect of each genetic covariate on quantile normalized SARA score, A‐T NEST score, age at first wheelchair use, and AFP levels using a linear mixed‐effects model (lmer function, R project *lme4* package^28^). For peripheral neuropathy, conjunctival telangiectasia, and malignancy, we used a generalized linear mixed‐effects model (glmer function, R project *lme4* package^28^) treating the response as a dichotomous variable. Associations between the neurological group and each genetic covariate were tested using a multinomial logistic regression (multinom function, R project *nnet* package^29^) with hospitals and families treated as fixed effects. For overall severity and eye movements responses, we used a mixed regression model for ordinal data, the ordered regression model implemented in the cmml function, R project *ordinal* package.^30^

## Results

### *Demographic Features and Employment*

The study included 57 individuals (23 males, 34 females) from 50 families (Supplementary Table 1). Mean age was 37.5 years (standard deviation [SD], 12.3; range 11–58), with 4 patients aged <18 years. Six women had offspring (5 from the UK cohort, 1 from the Dutch cohort). None of the men had offspring. Three individuals are deceased. Eleven individuals were in employment, and 2 were students. Seven had university degrees. Thirty‐nine patients were recruited from Papworth, 6 from Nottingham, and 12 from the Dutch cohort.

### *Neurological Features*

#### *Onset, Duration, Mobility, and Severity*

**Figure 1:**
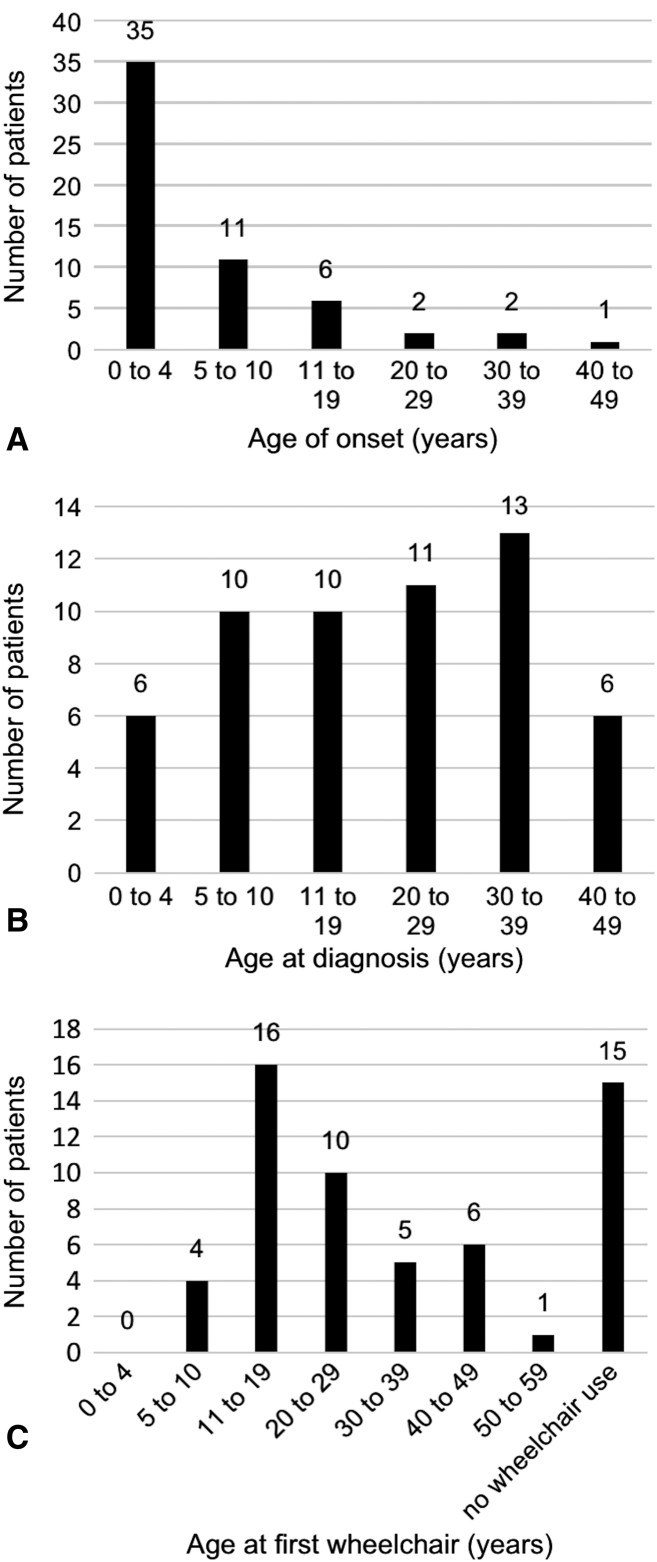
Age at (A) symptom onset, (B) diagnosis with ataxia‐telangiectasia, and (C) first using a wheelchair were obtained from each individual's clinical notes.

Age of onset is shown in Figure 1A. Most individuals (46 of 57) had their first symptoms by age 10 years. Individuals with disease onset before 10 years were considered to have early‐onset disease. Six had onset between 11 and 16 years. Five had adult onset disease.

Disease duration at last assessment ranged from 10 to 54 years. Disease duration was 10 to 20 years in 16 individuals, 20 to 30 years in 13, and > 30 years in 28.

Age at diagnosis ranged from 2 to 47 years (Fig 1B). The diagnosis was made within 1 year of symptom onset in 7 individuals and in 1 to 9 years in 17. There was a diagnostic delay of 10 to 20 years in 12 and > 20 years in 17 (not documented in 4).

Forty‐two individuals used a wheelchair and 15 were still ambulant. Median age at first getting a wheelchair was 20 years (range, 8–51; Fig 1C). Eleven individuals got their first wheelchair within 10 years of symptom onset, 17 in 10 to 20 years, and 14 after more than 20 years. Seven individuals were currently ambulant after a disease duration of 10 to 20 years and 8 after >20 years.

Seventeen individuals had mild disease, 26 had moderate disease, and 10 had severe disease. Classification of disease severity was not available for 4 individuals. There was 1 outlier in the severe group, a pediatric patient who used a wheelchair since age 8, and had an A‐T NEST score of 72.

#### *Neurological Phenotype*

Three distinct patterns of neurological deficits were observed. The neurological features and treatments in each group are summarised in Supplementary Table 2.

##### *Group A (Predominant Cerebellar Ataxia and/or Peripheral Neuropathy With Minimal or no Extrapyramidal Involvement)*

Nineteen individuals (33%) were in Group A. Severe oculomotor dyspraxia was common (11 of 19; 58%) and 6 patients had mild eye movement abnormalities (32%). A 15‐year‐old with mild disease had normal eye movements. The other patient with normal eye movements presented initially with a severe reaction to radiotherapy for breast cancer and had a mild phenotype consisting of peripheral neuropathy with only minimal limb ataxia.^31^ Disease severity was moderate or severe, except in the individual described above and 2 pediatric patients aged 11 and 15 years. Mean disease duration was 32 years.

##### *Group B (Disability Determined by a Mixture of Ataxia and/or Peripheral Neuropathy Plus Additional Extrapyramidal Features)*

Twenty‐eight individuals (49%) were in Group B. Their extrapyramidal features included dystonic posturing/neck dystonia (12 patients), dystonic tremor (11 individuals), choreiform movements (9 patients), and orofacial dystonia (5 patients). Two patients had a resting tremor. In this group, 12 of 28 (43%) exhibited prominent oculomotor dyspraxia, 43% had mildly abnormal eye movements, and 4 patients had normal eye movements. Disease severity was widely distributed. Six individuals had mild disease, 15 moderate disease, and 4 individuals were severely affected (not documented in 3). Mean disease duration was 30 years.

##### *Group C (predominantly extrapyramidal signs)*

Ten individuals (18%) were in Group C. One individual exhibited marked truncal dystonic spasms which limited his ability to walk.^32^ Another patient had a clinical phenotype typical of myoclonic dystonia, which improved markedly following deep brain stimulation.^33^ One individual had marked orofacial dystonia, but otherwise an essentially normal neurological examination with no limb ataxia or dystonia.^17^ One exhibited neck and limb dystonia, which was treated with botulinum toxin injections and another had hemichorea. Three siblings presented with severe resting tremor (onset between 16 and 34 years) and anterior horn neuronopathy.^18^ One individual presented with resting tremor at age 12 years and then developed chorea‐athetosis and dystonia. Another presented initially with chorea‐athetosis.

Two patients (20%) had marked oculomotor dyspraxia, 2 had mildly abnormal eye movements, and 6 of 10 of individuals had normal eye movements. Disease severity was classified as mild in 8 of 10, and 6 individuals in this group were in employment at the time of assessment. Mean disease duration was 28 years.

#### *Neurological Investigations*

##### *Neurophysiology*

Neurophysiology studies were performed in 34 individuals. Eighteen had an axonal sensorimotor polyneuropathy of varying severity. Three showed predominant involvement of the motor neurons. Nine had an axonal sensorimotor polyneuropathy with likely spinal muscular atrophy component. Four had normal/nearly normal neurophysiology studies.

Detailed neurophysiology results for 21 patients assessed through Papworth are shown in Supplementary Table 1.

##### *Radiology*

Brain MRI scans were available in a total of 35 individuals (including 23 performed through the Papworth service, 10 Dutch individuals, and 2 patients through the Nottingham clinic). Cerebellar atrophy was found in 29 individuals. Six scans were reported as showing a normal cerebellum (age, 19–46 years), of which one also showed atrophy of the left corpus nucleus caudatus. Seven individuals had white matter changes (single lesion in 2, a few punctate lesions in 5). Cerebral microbleeds were noted in 2 individuals. One had multiple, mainly in the occipital subcortical white matter (age 53). The other had a single microbleed in the right external capsule (age 55).

**Figure 2:**
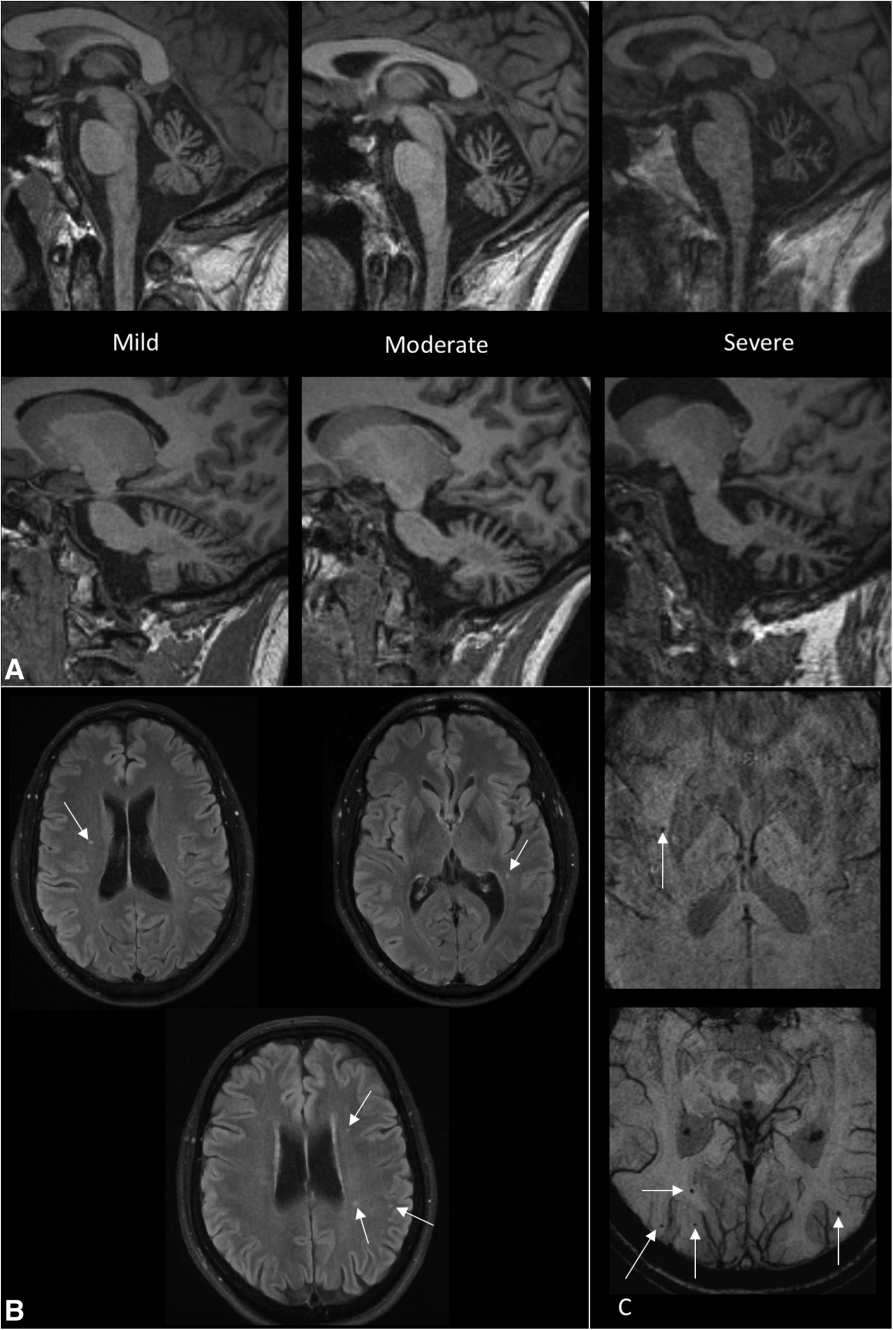
(A) MRI scans showing examples of mild, moderate and severe cerebellar atrophy. The top row shows midline images to assess vermian atrophy and the bottom row shows parasagittal images for assessing hemispheric atrophy. (B) MRI scans showing examples of white matter lesions. (C) MRI scans showing microbleeds. MRI = magnetic resonance imaging.

Brain MRI scans performed through Papworth were reviewed by a consultant radiologist. Cerebellar atrophy was observed in 23 of 23 scans. Atrophy of the cerebellar vermis was classified as mild in 8, moderate in 9, and severe in 6. Atrophy of the cerebellar hemispheres was classified as mild in 11, moderate in 7, and severe in 5 (Fig 2A–C).

### *Other Clinical Features*

#### *Endocrine function*

Mean height percentile was 48.1 (SD, 31.1; 53 patients evaluated). Mean BMI was 23.3 (SD, 4.8; 49 patients evaluated). Six individuals were underweight (BMI, <18.5), and 4 had gastrostomy feeding because of dysphagia and/or poor appetite. Two patients had type 2 diabetes. None had type 1 diabetes.

#### *Conjunctival Telangiectasia*

Conjunctival telangiectasia was observed in 35 of 56 individuals with documented assessment (63%).

#### *Respiratory Function*

No patient had severe lung disease. CT thorax was performed in 39 patients. Thirty showed normal results, 6 showed mild bronchiectasis, 1 showed mild emphysema (ex‐smoker), 1 showed patchy air trapping, and 1 showed two left upper lobe nodules (likely intrapulmonary lymph nodes).

Arterial blood gases showed mean PaO_2_ 12.4 kPa (SD, 1.34) and PaCO_2_ 5.17 kPa (SD, 0.52). Of 39 results, only one was abnormal with mild hypoxemia. Overnight oximetry in 37 subjects did not show hypoventilation, with mean oxygen saturations of 96.4% (SD, 1.35) and mean 4% desaturation index of 3.8 (SD 3.26). Pulmonary function data from 42 individuals showed mean FEV_1_ 80.2% (SD, 18.7), FVC 79.7% (SD, 21.6), and KCO 95.5% (SD, 19.3%). Twenty spirometries were restrictive; however, patients struggled with spirometric technique.

#### *Immunological Function*

Infection history was assessed in all patients and showed episodes of severe chickenpox (2), sinusitis (1), recurrent boils (1), frequent urinary tract infections (1), and sore throats 12 times per year (1). Two individuals were taking azithromycin prophylaxis for lower respiratory tract infections. No individuals were receiving immunoglobulin therapy.

Subtle signs of immune dysregulation and possible immune deficiency were observed in a number of patients. Immunoglobulin results were available in 52 patients. Polyclonal increases were observed in IgG (1), IgA (7), and IgM (12). One further patient had increased IgG and IgA. Four patients had a monoclonal gammopathy of unknown significance. One individual had mild panhypogammaglobulinemia. A slight reduction in total IgG was observed in 1 and IgA in 5 individuals. One had IgA deficiency (<0.07 μg/ml). IgE was below level of detection in 8 patients.

Lymphocyte subpopulation results were available in 44 patients. Slight reductions in CD4 and CD8 counts were found in 2 and 12 patients, respectively, and 16 patients had CD19 counts just below the normal range.

#### *AFP Levels*

Mean serum AFP was 176 μg/l (range, 2–600; SD, 146) in 45 individuals with a result available. Three (6.6%) had levels in the normal range

#### *Liver Ultrasound, LFTs*

Liver ultrasound was performed in 36 patients. Seven showed fatty liver and three detected liver lesions (hemangioma, neuroendocrine tumour, and metastatic prostate cancer). Fifteen of 44 patients with LFT results available had mildly abnormal results.

### *Malignancy*

Fourteen patients have been diagnosed with a malignancy. Solid tumours included five female unilateral estrogen‐receptor positive breast cancers (age, 28–44 years), one dermatofibrosarcoma protuberans of the breast (age 29), one neuroendocrine tumor (age 48), one pancreatic cancer (died age 48), one pelvic mass (possible germ‐cell tumor at age 11), and one prostate cancer (age 52). Lymphoid malignancies included T‐cell non‐Hodgkin's lymphoma (age 2), acute lymphoblastic leukemia (age 9), chronic myeloid leukemia (age 39), and chronic lymphoblastic leukemia (patient also had breast cancer and died aged 47 years). One patient died of acute lymphoblastic leukemia aged 51.

### *Genetics*

#### *Family History*

Nineteen individuals had a family history of ataxia‐telangiectasia in a sibling, and 4 had affected siblings who died from malignancies (primary hepatoma, ectopic pituitary tumor, and 2 with lymphomas). There was parental consanguinity in two families. Twenty of 114 parents had been diagnosed with a cancer (18%). Mothers of 10 patients developed breast cancer (aged 39–64 years).

#### *Cytogenetic Features*

Chromosomal radiosensitivity was normal in 15 of 48 individuals tested (31%) and raised in 33 of 48 (69%). Two had a high level, the same as expected in classic ataxia‐telangiectasia.

#### *Mutation Analysis*

Mutation analysis (Supplementary Table 1) revealed mutations in 111 of 114 alleles. Patients were classified based on the mutation present causing the retained kinase activity.

Individuals in Genetic Group 1 all have a leaky splice site mutation, which allows some normal ATM protein to be expressed with kinase activity. Fourteen patients in this group have the c.5763‐1050A > G mutation.

Individuals in Genetic Group 2 all have a missense mutation, which results in the presence of a mutant protein with some ATM kinase activity.

Individuals in Genetic Group 3 have mutations affecting the initiator methionine codon. These mutations allow expression of a very low level of truncated protein.^31^ Patients with these mutations have been noted to have a milder clinical course,^24^ and there is some uncertainty of ATM activity associated with these mutations.

Individuals in Genetic Group 4 have one confirmed mutation and one mutation which has not been identified or not been completely characterized. Patient 50 has a missense mutation Val2716Ala which is associated with retained ATM activity. The other 6 patients showed a low level of ATM protein and low level of ATM kinase activity. Complementary DNA (cDNA) was examined in these cases. Two have alterations found at the cDNA level, but no potentially causative mutations found in genomic DNA. The mutations giving protein with activity are likely to be intronic splice site mutations.

Twelve patients (1, 2, 18a,b, 26, 31, 33, 35 and 37a‐c, 41) had two “milder” mutations, which are both known to be associated with retained ATM kinase activity.

#### *Genotype‐Phenotype Correlation*

Cell lines from 51 of 51 patients tested showed retained expression of some ATM protein. This ranged from just detectable (~5%) to near normal.

ATM kinase activity was tested in cell lines from 45 patients. Cells derived from patients 34 and 42 showed possibly a low level of ATM kinase activity.

The 4 patients (34, 42, 43, and 44) with absent or possibly just detectable ATM kinase activity had more severe disease. Onset of symptoms was between 1 and 2.5 years, with first wheelchair use at 8 years in 2 patients and 11 and 13 years in the other 2. Two have percutaneous endoscopic gastrostomy feeding tubes.

There are two major subgroups of variant patients: those with a missense mutation producing some ATM protein with retained activity (Group 2, and 1 in Group 4) and those with a leaky splice site mutation allowing expression of a low level of normal ATM with some activity (Group 1, and most likely 6 in Group 4).

**Figure 3:**
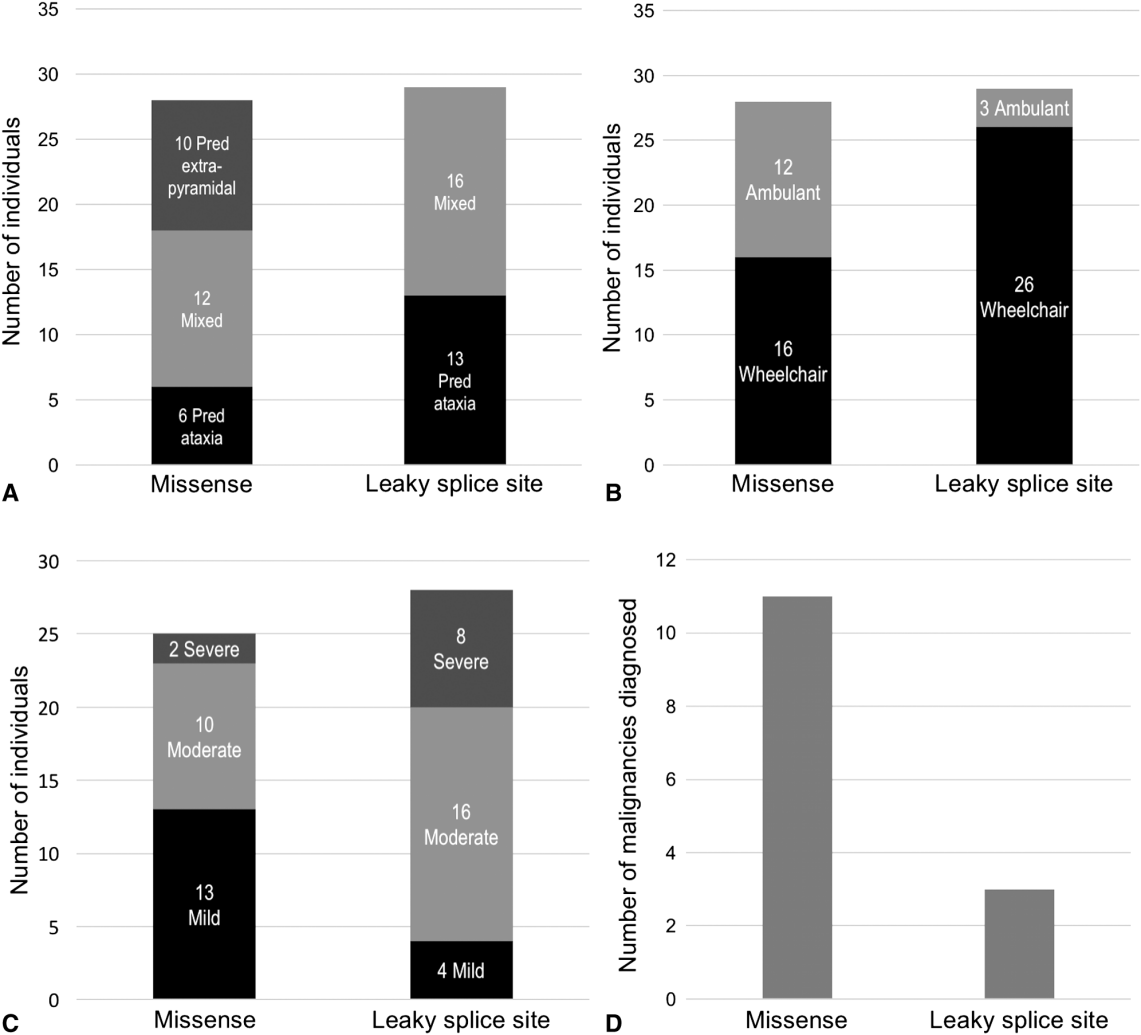
(A) Graph shows number of individuals in each neurological phenotypic group in patients with retained ATM kinase activity attributed to a missense mutation or a leaky splice‐site mutation. (B) Graph shows numbers of individuals who use a wheelchair or are still ambulant in patients with retained ATM kinase activity due to a missense mutation or a leaky splice‐site mutation. (C) Graphs shows cross‐sectional clinical neurological disease severity in patients with retained ATM kinase activity attributed to a missense mutation or a leaky splice‐site mutation. (D) Graph shows number of malignancies diagnosed in patients with retained ATM kinase activity attributed to a missense mutation or a leaky splice‐site mutation. ATM = ataxia telangiectasia‐mutated.

Individuals with at least one missense mutation which produces a mutant protein with residual kinase activity had milder neurological disease compared to the rest of the cohort who have leaky splice site mutations or mutations in the initiator methionine codon. There were statistically significant differences in overall severity (*p* = 0.0256; odds ratio [OR] confidence interval [CI] = [0.0265, 0.7901]) and age at first wheelchair use (*p* = 0.0012; CI = [0.2694, 1.0926]), and they were more likely to have normal eye movements (*p* = 0.0011; OR CI = [0.0333, 0.4315]). These individuals were also more likely to have a malignancy (*p* = 0.0389), with an odds ratio of 4.94 (OR CI = [1.0849, 22.5292]) compared with the rest of the cohort (see Fig 3; Supplementary Table 3 and Supplementary Table 2).

Although there were no statistically significant differences in neurological group, it is striking that predominant extrapyramidal signs were only observed in patients with missense mutations (Group 2 and patient 50).

Individuals with a high ATM protein level had lower AFP levels (*p* = 0.0235; CI = [–1.7628, –0.1270]), and slightly higher A‐T NEST scores (*p* = 0.0319; CI = [0.0713, 1.5825]).

There were no statistically significant associations between chromosomal radiosensitivity or number of mild mutations with neurological or other clinical features.

## Discussion

This study describes the largest published cohort of individuals with variant ataxia‐telangiectasia who have undergone detailed genetic and clinical evaluation. We show that all individuals included in this survey have neurological involvement, while systemic features are mild or absent, apart from an increased risk of malignancy.

Our study confirms that disease severity in variant ataxia‐telangiectasia is milder compared to the classic form. Our cohort includes 31 patients aged > = 40 years, 26% were still ambulant, 6 had children, and 11 were in paid or voluntary employment. Consistent with other reports,^13^ the majority of individuals included in this study had their symptom onset in early childhood. However, disease progression is then much slower than in the classic form.

We formally graded disease severity according to a combination of neurological rating scales (A‐T NEST, SARA) and assessment of mobility and self‐care. According to this grading, 32% of patients had mild disease and only 19% severe disease. Similar to multiple sclerosis, another multisystem neurological disease, we have chosen to grade disease severity mainly according to functional impairment and mobility.^34^ Further studies will be required to validate our severity rating.

All individuals in our cohort showed neurological abnormalities on clinical examination. A range of neurological systems were affected (cerebellum, peripheral nerves, eye movements, and extrapyramidal system), but none universally. For example, 10 individuals had no significant cerebellar features, 11 had normal eye movements, and 8 had no evidence of peripheral nerve involvement. A large proportion had extrapyramidal symptoms, and in some, extrapyramidal presentation was the predominant or only clinical feature. The most common extrapyramidal symptoms were dystonia and dystonic tremor. Chorea and parkinsonism were rare. Presence of exclusive extrapyramidal symptoms has not been reported in classic A‐T. This suggests possible differences in neuronal vulnerability and pathways between the classic and variant form of the disease and could be further explored with imaging studies.

With the limitation of small sample size, our study suggests that disease severity is milder in individuals with a purely extrapyramidal presentation compared to those who have ataxia. This finding could partly reflect effective treatments (botulinum toxin, medication, and deep brain stimulation) for extrapyramidal symptoms. Longitudinal studies will be important in order to evaluate whether individuals with exclusive extrapyramidal or cerebellar symptoms eventually progress to develop a more mixed presentation.

Our study is the first to report on neurological investigations in a large number of patients with variant ataxia‐telangiectasia. We found that cerebellar and vermian atrophy was the most common imaging abnormality, although 5 individuals had a normal cerebellar appearance. Cerebral microhemorrhages were shown in 2 of 19 patients whose scan included gradient echo and SWI and white matter abnormalities occurred in 7 of 23 patients. Reported radiological findings in classic ataxia‐telangiectasia include cerebellar atrophy and multiple punctate hemosiderin deposits, but in some individuals also very extensive white matter abnormalities,^35^ which were speculated to represent “edema from vascular leakage.”^36^ It is possible that that the extracerebellar abnormalities we observed could represent an earlier stage of a process that progresses more floridly in some individuals with classic ataxia‐telangiectasia.

Neurophysiology studies showed that most patients tested had an axonal sensorimotor polyneuropathy (18 of 34); 3 had predominant anterior horn involvement, and 9 had a combination of both. Interestingly, neurophysiology was normal in 4 included individuals, all of whom had mild disease progression but otherwise a mixture of associated neurological symptoms.

Our study confirms that the risk of malignancy is significantly increased in variant ataxia‐telangiectasia. The cancers in our cohort were predominantly female premenopausal breast cancer and hematological malignancies. There were only two childhood tumors, which is consistent with Reiman's study^24^ which showed a protective effect of retained ATM kinase activity against childhood tumors.

Individuals with a missense mutation which causes production of a mutant ATM protein with retained kinase activity were approximately 5 times more likely to have a malignancy than individuals in the other genetic groups. It has been previously reported that a specific missense mutation, c. 7271T > G, p.(Val2424Gly), causes a high risk of breast cancer.^16^ Canadian Mennonites who were homozygous for the c.6200 C > A (p. Ala2067Asp) missense mutation also showed a high risk of malignancy.^14^ This is consistent with the idea that specific missense mutations cause a high risk of malignancy, which suggests a possible gain of function mechanism.

We found that individuals with a missense mutation have milder neurological features, as measured by overall severity and A‐T NEST score. These patients are more likely to remain ambulant and more likely to have normal eye movements.

The 4 individuals (34, 42, 43, and 44) who have borderline low/absent ATM kinase activity had more severe neurological features, and 2 required gastrostomy feeding. Patients with a mutation in the initiator methionine were included because of the evidence of longevity in some of these patients and that their cells express a low level of N‐terminally truncated ATM protein, although, as it turns out, without measurable activity using the assay described. A more sensitive assay would be helpful in detecting differential low levels of ATM activity/signaling associated with different ATM proteins. This may also be true of some ATM missense mutations (eg, c.5228C > T; p.(Thr1743Ile) where current assays do not detect any activity associated with the expressed protein. It is possible that there may be sufficient activity retained to have some positive clinical effect. Individuals (3, 4, 5, 16, 20, 22, 23a, 23b, 27, 31, and 32) with a high ATM level (around 50% or more) showed higher A‐T NEST scores (less severe disease) and lower AFP levels. Taken together, these results indicate that the reduced severity of the neurological features may reflect the level of ATM kinase activity, which is consistent with the fact that classic ataxia‐telangiectasia (where ATM kinase is absent) generally presents with a more severe phenotype than variant ataxia‐telangiectasia.

Each of the five sets of siblings included here showed similar neurological features. For example, 3 affected sisters all have ataxia together with peripheral neuropathy, dystonic tremor, and essentially normal eye movements. Three Dutch siblings all presented with resting tremor and distal muscle weakness attributed to anterior horn cell involvement.^18^ This similarity between siblings is consistent with the fact that the neurological phenotype is partly determined by shared “genetic background.” However, we did not find significant genotype‐phenotype association with regard to neurological group in the cohort overall, indicating an additional influence of shared environmental factors or disease modifying genes.

Systemic complications were mild or absent, apart from the increased risk of malignancy. This is consistent with previous reports^4^, ^13^, ^14^ and has significant implications on surveillance and management of these patients. None of the individuals in this study had significant respiratory disease. Functional testing with arterial blood gases and overnight oximetry was essentially normal. Even though 42 of 53 tested individuals had a detectable abnormality on immunological testing, in general this was subtle and of doubtful clinical significance. No individual required treatment with immunoglobulin replacement, and only 2 used prophylactic antibiotics for recurrent pulmonary infections. Our cohort includes only 2 patients with diabetes mellitus, which suggests a similar prevalence to in the general population. However, more than one quarter of included individuals had abnormalities in their liver function tests, the relevance of which needs to be further investigated in future studies.

Our results suggest that variant ataxia‐telangiectasia is likely to be under‐ or misdiagnosed. The time from first symptoms to diagnosis was over 10 years in more than half of our cohort, and we included individuals with only very mild neurological symptoms. Clinicians may not be familiar with the wide range of clinical presentations of variant ataxia‐telangiectasia, where eye movements can be normal, conjunctival telangiectasia absent, neurological presentation mainly extrapyramidal, and AFP levels within normal range.

Individuals with variant ataxia‐telangiectasia require surveillance for malignancy and management guidance—including breast screening in women, minimising exposure to ionising radiation and facilitating specialist assessments. Missense mutations are associated with milder neurological presentations, but have a particularly high malignancy risk, and it is important for clinicians to be aware of these phenotypes.

## Author Contributions

K.S., N.O., H.B., M.W., M.T., A.M.R.T., and A.H. contributed to the conception and design of the study. All authors contributed to the acquisition and analysis of data. K.S., N.v.O., N.O., H.B., D.S., J.R., L.B., M.T., A.M.R.T., and A.H. contributed to drafting the text and preparing the figures.

## Potential Conflicts of Interest

Nothing to report.

## Supporting information

Supporting Information Tables
