# Supplementary material for: Genotype, extrapyramidal features, and severity of variant ataxia‐telangiectasia
Source: Ann Neurol. 2019 Jan 29;85(2):170–80. doi: 10.1002/ana.25394 (PMC6590299; doi:10.1002/ana.25394)
Supplement: Supplementary file 1 — Supporting Information Tables [file ANA-85-170-s001.pdf]

SUPPLEMENTARY TABLE 1. ***ATM*** **Mutations, Protein Changes, and ATM Kinase Activity in 57 Variant Ataxia-Telangiectasia Patients**

Patient (sex, age)	ATM Mutation 1	ATM Mutation 2	Neur
Genetic group 1: some normal ATM protein, with residual kinase activity present, attributed to a leaky splice site mutation			
1 (F,38)	c.5763-1050A > G, p.(Pro1922fs)	duplication of ATM exons 53-61	B
2 (M,41) (Ref 21)	c.5763-1050A > G, p.(Pro1922fs)	c.5763-1050A > G, p.(Pro1922fs)	A
3 (F,36)	c.5763-1050A > G, p.(Pro1922fs)	c.9022C > T, p.(Arg3008Cys)	B
4 (M,47)	c.5763-1050A > G, p.(Pro1922fs)	c.9022C > T, p.(Arg3008Cys)	B
5 (F,55)* (Ref 15)	c.5763-1050A > G, p.(Pro1922fs)	c.9139C > T, p.(Arg3047Ter)	B
6 (F,55)	c.5763-1050A > G, p.(Pro1922fs)	c.2T > C, p.(Met1Thr)	B
7 (F,20)	c.5763-1050A > G, p.(Pro1922fs)	c.1563_1564delAG, p.(Glu522IlefsTer21)	B
8 (F,40)*	c.5763-1050A > G, p.(Pro1922fs)	c.6136delC, p.(Leu2046fsTer1)	A
9 (M,38)*	c.5763-1050A > G, p.(Pro1922fs)	c.6199-6_6227del35, p.(Ala2067GlnfsTer10)	B
10 (F,50)	c.5763-1050A > G, p.(Pro1922fs)	c.8418 + 2_5delTGAG, p.(Val2757_Met2806del)	B
11 (M,49)	c.5763-1050A > G, p.(Pro1922fs)	c.8786 + 1G > A, p.(Gly2891fs)	A
12 (M,21)	c.5763-1050A > G, p.(Pro1922fs)	c.8491_8497del7, p.(Phe2831ThrfsTer24)	B
13 (M,60)*	c.5763-1050A > G, p.(Pro1922fs)	c.2284-2285delCT, p.(Leu762fs)	A
14 (F,22)*	c.5763-1050A > G, p.(Pro1922fs)	c.6198 + 1G > A, p.(Leu2033ProfsTer15)	B
15 (M,31)	c.8418 + 681A > G, p.(Glu2807ValfsTer4)	c.1564_1565delGA, p.(Glu522llefsTer43)	B
16 (M,45)	c.1066-6T > G, Loss of exon 9	c.9023G > A, p.(Arg3008His)	A
17 (F,31)	c.6807G > A, Loss of exon 46	c.1158delG, p.(Lys387fs)	B
18a (F,43), 18b (M,40)	c.331 + 5G > A	c.331 + 5G > A, exon 4 splice donor defect	A, A
19 (F,48)* (Ref 4,7)	c.496 + 5G > A, p.Arg111_Glu166del55insLys	c.7875_7876delTGinsGC, p. Asp2625_Ala2626delinsGluPro	B
Genetic group 2: mutant ATM protein present with residual kinase activity from a missense mutation			
20 (F,27)	c.7271T > G, p.(Val2424Gly)	c.103C > T, p.(Arg35Ter)	C
21 (F,48)*	c.7271T > G, p.(Val2424Gly)	c.8266A > T, p.(Lys2756Ter)	B
22 (M,26)	c.7271T > G, p.(Val2424Gly)	c.3G > T, p.(Met1Ile)	A
23a (M,47), 23b (M,36)	c.7271T > G, p.(Val2424Gly)	c.8269-loss of exon 57 by MLPA	A, A
24 (M,41) (Ref 33)	c.743G > T, p.(Arg248Leu)	c.8266A > T, p.(Lys2756Ter)	C
25 (M,52) (Ref 32)	c.743G > T, p.(Arg248Leu)	c.5623C > T, p.(Arg1875Ter)	C
26 (F,27) (Ref 17)	c.590G > A, p.(Gly197Glu)	c.590G > A, p.(Gly197Glu)	C
27 (F,56c) (Ref 31)	c.8672G > A, p.(Gly2891Asp)	c.1A > G, p.(Met1Val)	A
28 (F,25)	c.8480T > G, p.(Phe2827Cys)	c.1564_1565delAG, p.(Glu522IlefsTer21)	B
29a (F,50c), 29b (F,47c), 29c (F,41c)	c.7184A > T, p.(Asp2395Val)	c.6490G > T, p.(Glu2164Ter)	BBB

SUPPLEMENTARY TABLE 1. Continued

Patient (sex, age)	ATM Mutation 1	ATM Mutation 2	Neur
30 (F,32)	c.875C > T, p.(Pro292Leu)	c.5129_5763-1060del9263 p.(Glu1669AspfsTer16) deletion of exons 35 to 38	A
31 (M,21)	c.875C > T, p.(Pro292Leu)	c.8494C > T, p.(Arg2832Cys)	B
32 (M,32)	c.6115G > A, p.(Glu2039Lys)	c.8609_8610delAT, p.(Asp2870GlufsTer10)	C
33 (F,11)	c.9103C > T, p.(Leu3035Phe)	c.9103C > T, p.(Leu3035Phe)	A
34 (M,13)(PD)	c.7013T > C, p.(Leu2338Pro)	c.6056A > G, p.(Tyr2019Cys)	B
35 (M,15)	c.8494C > T, p.(Arg2832Cys)	c.1844C > T, p.(Leu615Pro)	B
36 (F,40) (Ref 4,7)	c.2909T > G, p.(Leu970Arg)	c.6908dupA, p.(Glu2304fs)	B
37a (M,39), 37b† (M,48), 37c† (M,48)	c.3136C > T, p.(Leu1046Phe) (Ref 4,7,18)	c.7622T > G, p.(Leu2541Arg)	CCC
38 (F,42) (Ref 4,7)	c.8147T > C, p.(Val2716Ala)	c.5932G > T, p.(Glu1978Ter)	C
39† (F,47)* (Ref 4,7)	c.8147T > C, p.(Val2716Ala)	c.1391_1395delTGTGT, p.(Leu464SerfsTer21)	B
40 (F,47)* (Ref 4,7)	c.8147T > C, p.(Val2716Ala)	c.717_720delCCTC, p.(Val240fs)	B
41 (F,58c) (Ref 4,7)	c.8147T > C, p.(Val2716Ala)	c.2922-1G > A, p.(Asn975_Trp1026del)	B
Genetic group 3: patients with mutations in initiator methionine, with no likely kinase activity			
42 (F,23)(PD)	c.2T> C, p.(Met1Thr)	c.9139C > T, p.(Arg3047Ter)	B
43 (F,42)*	c.2T> C, p.(Met1Thr)	c.640_640delT, p.(Ser214ProfsTer16)	A
44 (M,22)*	c.2T> C; p.(Met1Thr)	c.7665delinsGTGA, p.His2555GlnfsX2	A
Genetic group 4: patients with residual ATM kinase activity with one mutation not detected or incompletely resolved			
45 (F,30)	c.5644C > T, p.(Arg1882X)	Not detected	B
46a (M,56), 46b (M,54)	c.170G > A, p.(Trp57Ter)	c.3403del174(del exon 24), incompletely resolved	A, A
47 (F,26)	c.387delA, p.(Asp130fsTer23)	Skipping exons 31 and 32, incompletely resolved	B
48 (F,31c)	c.2466 + 6 T > A complex, unresolved	Not detected	A
49 (F,15)	c.2731dupG, p.(Ala911GlyfsTer9)	Possible splice mutation involving exons 33 and 34	A
50 (F,45)* (Ref 4,7)	c.8147T > C, p.(Val2716Ala)	Not detected	C

Ref = reference to previous publication, † = individual deceased, c = individual has offspring, * = kinase activity not tested, Neur = neurology group, PD = possibly just detectable kinase activity.
ATM = ataxia telangiectasia-mutated.

SUPPLEMENTARY TABLE 2. Neurological Features and Malignancies in Each Neurological Phenotypic Group

	A: Cerebellar Ataxia and/or Peripheral Neuropathy With Minimal or No Extrapyramidal Involvement	B: Cerebellar Ataxia and/or Peripheral Neuropathy Plus Additional Extrapyramidal Features	C: Extrapyramidal Signs Without Significant Ataxia and/or Peripheral Neuropathy
No. of individuals	19	28	10
Mean age at assessment	38.8 years (range, 11–56)	35.7 years (range, 13–56)	39.8 years (range, 27–52)
Mean disease duration (yr)	32	30	28
Onset	Early 16: Late 3	Early 25: Late 3	Early 5: Late 5
Progression	Slow: 5	Slow: 9	Slow: 7
	Slow/Mod: 4	Slow/Mod: 2	Slow/Mod: 1
	Moderate: 7	Moderate: 9	Moderate: 1
	Rapid: 3	Rapid: 8	Rapid: 0
Current severity	Mild: 3	Mild: 6	Mild: 8
	Moderate: 10	Moderate: 15	Moderate: 1
	Severe: 6	Severe: 4	Severe: 0
		Not documented: 3	Not documented: 1
Mean A-T NEST	61.0 (range, 36–95)	58.2 (range, 34–87)	64.3 (range, 57–72)
Mean SARA	26.0 (range, 4–38)	22.1 (range, 6–34)	11.6 (range, 6–21)
Eye movements	Normal: 1	Normal: 4	Normal: 6
	Mildly abnormal: 6	Mildly abnormal: 12	Mildly abnormal: 2
	Moderate/severe oculomotor dyspraxia: 11	Moderate/severe oculomotor dyspraxia: 12	Moderate/severe oculomotor dyspraxia: 2
Neurophysiology	N = 12	N = 14	N = 8
	Normal neurophysiology studies: 1	Axonal sensorimotor neuropathy: 10	Normal neurophysiology studies: 1
	Axonal sensorimotor neuropathy: 8	Axonal sensorimotor neuropathy and likely/ possible spinal muscular atrophy: 4	Axonal sensorimotor neuropathy: 3
	Axonal sensorimotor neuropathy and likely spinal muscular atrophy: 3		Axonal peripheral neuropathy and affected motor neurons: 3
			Affected motor neurones only: 1
Neurological treatment	No specific neurological treatment relating to A-T: 14	No specific neurological treatment relating to A-T: 16	No specific treatment relating to A-T: 6

SUPPLEMENTARY TABLE 2. Continued

	A: Cerebellar Ataxia and/or Peripheral Neuropathy With Minimal or No Extrapyramidal Involvement	B: Cerebellar Ataxia and/or Peripheral Neuropathy Plus Additional Extrapyramidal Features	C: Extrapyramidal Signs Without Significant Ataxia and/or Peripheral Neuropathy
	Amitriptyline for painful neuropathy: 1	Single agents:	Deep brain stimulation: 1
	Propranolol and primidone for tremors: 1	Madopar: 1	Botulinum toxin injections: 1
	Clonazepam and baclofen: 1	Trihexiphenidyl: 2	Botulinum toxin injections and clonazepam: 1
	Amitriptyline and oxybutynin: 1	Sinemet plus: 2	Baclofen and benzhexol: 1
		Baclofen: 1	
		Oxybutynin (for drooling): 1	
		Leviteracitam: 1	
		Two agents:	
		Propranolol and primidone: 1	
		Pregabalin and amantadine: 1	
		Amifampridine and trihexiphenidyl: 1	
		Four agents:	
		clonazepam, amantadine, pregabalin, trihexyphenidyl	
Malignancy	Breast cancer 43y	Breast cancers 29y, 43y	Breast cancers 28y, 33y
	Dermatofibroma 29y	Neuroendocrine tumour 48y	Prostate cancer 52y
	Germ cell tumour 11y	T-cell non-Hodgkin's lymphoma 2y	Pancreatic cancer died 48y
	ALL 9y	CLL died 47 years	ALL died 51 years
		CML 39y	

ALL = acute lymphoblastic leukemia; A-T NEST = Ataxia-Telangiectasia Neurological Examination Scale Toolkit; CLL = chronic lymphocytic leukemia; CML = chronic myeloid leukemia; SARA = Scale for Assessment and Rating of Ataxia.

SUPPLEMENTARY TABLE 3. Comparison of Clinical Features in Individuals Either With One or More Missense Mutations Which Produce Mutant Protein With Residual Kinase Activity or With a Leaky Splice Site Mutation Also Expressing ATM Protein With Activity

	> = 1 Missense Mutation Producing Mutant Protein With Retained ATM Kinase Activity<br>28 individuals	Leaky Splice Site Mutation Producing Protein With Retained ATM Kinase Activity (1)<br>29 individuals
**Demographic features**		
Male/female	11/17	12/17
Mean age (range)	38 (13–58)	37 (15–56)
Median age at onset (range)	3 (0.5–44.0)	3 (1–22)
Median age at diagnosis (range)	28 (2–47)	16 (3.5–44.0)
**Neurological features**		
Neurology group A/B/C	6/12/10	13/16/0
No. with wheelchair/still ambulant	16/12	26/3
Median age at first wheelchair among users (range)	30.5 years (8–51)	16 years (8–40)
Cross-sectional Clinical Neurological Disease Severity (mild/moderate/severe)	13/10/2 (not recorded in 3)	4/16/8 (not recorded in 1)
Median SARA	17	24.5
Median AT-NEST	61.5	56
Peripheral neuropathy (present/absent)	21/5 (not recorded in 2)	25/3 (not recorded in 1)
Eye movements (moderate-to-severe oculuotor dyspraxia/mild abnormalities/normal)	4/13/11	21/7/1
**Non-neurological features**		
Conjunctival telangiectasia (present/absent)	14/12 (1 not documented)	21/8
CT chest	Bronchiectasis 3	Bronchiectasis 3
	Normal findings 9	Normal findings 21
	intrapulmonary nodules 1	Emphysema 1
	Not done 12	Patchy air trapping 1
		Not done 3
Personal history of malignancy	Breast cancer (4 patients)	Breast cancer
	CML	T-cell ALL
	CLL	Dermatofibrosarcoma protuberans
	ALL	
	Non-Hodgkin's lymphoma	
	Pancreatic cancer	
	Germ-cell tumor	
	Prostate cancer	

SUPPLEMENTARY TABLE 3. Continued

	> = 1 Missense Mutation Producing Mutant Protein With Retained ATM Kinase Activity<br>28 individuals	Leaky Splice Site Mutation Producing Protein With Retained ATM Kinase Activity (1)<br>29 individuals
**Laboratory features**		
Mean alpha-fetoprotein, μg/l (range)	203 (10–600)	118 (2–332)
Chromosomal radio-sensitivity (raised/normal)	16/6 (6 not done)	17/9 (3 not done)

These also include the 3 group 3 patients.

ALL = acute lymphoblastic leukemia; ATM = ataxia telangiectasia-mutated; A-T NEST = Ataxia-Telangiectasia Neurological Examination Scale Toolkit; CLL = chronic lymphocytic leukemia; CML = chronic myeloid leukemia; CT = computed tomography; SARA = Scale for Assessment and Rating of Ataxia.
